# Correlates of regret with treatment decision-making among Japanese women with breast cancer: results of an internet-based cross-sectional survey

**DOI:** 10.1186/s12905-019-0783-5

**Published:** 2019-07-02

**Authors:** Keiko Yamauchi, Motoyuki Nakao, Mitsuyo Nakashima

**Affiliations:** 1 Department of Public Health, School of Medicine, Kurume University, 67 Asahimachi, Kurume, Fukuoka, 830-0011 Japan; 2 Department of Nursing, School of Medicine, Fukuoka University, Fukuoka, Japan

**Keywords:** Breast cancer, Japanese women, Decision-making, Decisional regret, Decisional role

## Abstract

**Background:**

Satisfaction with medical decisions among patients with cancer is associated not only with the results of decisions they make but also with how they make those decisions. To elucidate the decision-making process among Japanese women with breast cancer, we explored the correlates of regret with patients’ treatment decision-making.

**Methods:**

An Internet-based cross-sectional survey was utilized. Japanese women (*N* = 467) who self-reported that they had been diagnosed with stage 0­II breast cancer participated. Data regarding their decisional role (active, collaborative, or passive) in treatment decision-making, their most regrettable experience regarding their decision-making, the importance of various factors related to decision-making at the time, and clinical and sociodemographic factors were obtained. A forced-entry logistic regression analysis was performed on the likelihood that patients would have some regrets regarding the decision-making process.

**Results:**

About half the women expressed some regret (51.4%). Women who had a mastectomy were significantly more likely to have regret than women who had breast conserving surgery. Correlates of regret differed by surgical type. For women who had a mastectomy, those who were aged ≥50 years when diagnosed, or who made their decisions collaboratively with their doctors were significantly less likely to have regret with the decision-making. For women who had breast conserving surgery, those who worked on a contract or part-time basis or whose decision-making roles matched their preferred role were significantly less likely to have regret. Among women who reported some regret, 23.8% expressed that their most regrettable experience concerned gathering information, while 21.3% regretted not consulting with others. For women who were diagnosed at a younger age, the influence on their sex life and pregnancy and childbirth was more important when making their treatment decisions than for women diagnosed an older age.

**Conclusions:**

Approximately half of the Japanese women with breast cancer in this study reported some regret in the treatment decision-making process. Effective participation in decision-making differed by surgical types. Additionally, women who are diagnosed with breast cancer at a relatively younger age, as compared to those who are older, may need additional information and support regarding their sex life and fertility after cancer treatment.

**Electronic supplementary material:**

The online version of this article (10.1186/s12905-019-0783-5) contains supplementary material, which is available to authorized users.

## Background

Breast cancer (BC) is the most common type of cancer in Japan, and the number of women diagnosed with BC has recently increased; in 2017, about 89,000 Japanese women were diagnosed with BC [1]. These women are required to make difficult decisions regarding primary treatment after diagnosis such as whether to undergo a total mastectomy (TM) or breast conserving surgery (BCS) to resect primary tumors.

Satisfaction and regret with medical decisions as well as quality of life (QOL) among patients with BC are associated not only with the results of the decisions they make, but also with how they make those decisions; however, prior findings concerning the influence of this decision-making vary. There are discussions based on the level of involvement in decision-making. More active participation is associated with higher QOL [2, 3]; patients who collaborate with their physicians to make treatment decisions have less regret with treatment and higher QOL compared with patients who make decisions by themselves, or patients who entrust their physicians to determine the treatment [4, 5]. Other discussions concern the congruence of preferred and actual participatory styles. Decisional regret is lower and QOL is higher among patients whose involvement in decision-making is congruent with their preferred involvement, regardless of how they made treatment decisions [6–8].

Several studies have addressed the treatment decision-making process of Japanese women with BC, their preferred and perceived involvement in decision-making, and the impact of participation on satisfaction with their decision and the decision-making process [9–11]; however, these studies have not always reached consistent conclusions. For instance, the proportion of women who preferred to choose their cancer treatment collaboratively with their physicians ranged from 13 to 70% [9–11]. Participants were more satisfied with the treatment decision-making process when their participation in the process matched their preferred role [11]; however, another study found that almost all women (99%) were satisfied with the decision-making process regardless of congruence between their preferred and perceived participation, nor the role they played [9].

It should be noted, however, that studies conducted outside of Japan revealed that even patients with BC who were satisfied with their decisions reported some regrets with their decision-making process [3, 12]. This finding suggests that exploring decision-making regret might be helpful in gaining a better understanding of decision-making itself. The aforementioned studies involving Japanese women with BC focused on decision satisfaction or satisfaction with decision-making process [9–11]. We believe that exploring not only satisfaction but also regret with decision-making would provide new information regarding treatment decision-making for Japanese women with BC, which could be important in improving patients’ decisional satisfaction.

To better understand Japanese women’s decision-making for BC treatment, we investigated the extent that women experienced decision-making regret, the association of regret with decisional roles and other factors, and women’s most regrettable decision-making-related event. Furthermore, we examined the degree of importance of various clinical and social information for Japanese women when they considered their BC treatment.

## Methods

### Sample and data collection

The details of the study design and sample have been described in a previously published article and are reviewed herein [11]. We conducted a cross-sectional, self-reported Internet survey of Japanese females with BC/survivors using an online marketing research company (Rakuten Research Inc., Tokyo, Japan). We analyzed data from 475 women who completed the online questionnaire from March 11 to 14, 2016, and who reported that they had been diagnosed with stage 0–II BC. All data were complete.

### Measurements

Treatment decision-making role was elicited using the Japanese version of the Control Preference Scale [9]. Participants indicated the final decision-making role that best described their involvement in the decision-making process: active, “myself” and “myself with consideration of the opinions of the physician”; collaborative, “the physician and I together” and passive, “the physician” and “the physician with consideration of my opinions.” Their preferred role before diagnosis and their perceived role (actual role) in deciding treatment for their BC was elicited. Decisional congruence between preferred and actual roles was computed based on the five decision making styles.

Regret regarding the medical decision-making process was evaluated using the item, “have you had any regret or something you wanted to do over again in the treatment decision-making process?” Responses were made using a numerical rating scale: *having nothing to do over again* (0) to *everything to do over again* (100). Because of the skewed nature of the responses (48.8% reported *having nothing to do over again*), we dichotomized the variable (having no regret vs. having some regret). Additionally, women who had some level of regret expressed their most regrettable experience regarding their decision-making in areas such as choosing a hospital, building trusting relationships with doctors, gathering information, taking the time to consider options, and consulting with others.

We also assessed the degree of importance of various clinical information for Japanese women when they considered their BC treatment, such as cancer characteristics, treatment benefits and risks, obtaining a second opinion, and medical expenses, as well as life influences such as pregnancy and childbirth, sexual activity, and work/housework. Participants rated each item on a 5-point scale (1 = not at all to 5 = very).

### Statistical analyses

Descriptive statistical analyses were used to determine participants’ characteristics and their most regrettable decision-making experiences. A forced-entry logistic regression analysis was conducted with whether or not participants had some regrets as the dependent variable. Independent variables included demographic variables (i.e., current age, age at diagnosis, marital status, educational level, household income, and employment status) and clinical variables (i.e., time since diagnosis, cancer stage, surgery type, whether they received radiation, chemotherapy and/or hormone therapy), preferred and perceived decision-making roles, and congruity between preferred and perceived decision-making role. Adjusted odds ratio (AOR) were reported together with 95% confidence intervals. Chi-square tests were performed to examine proportional differences between age groups upon diagnosis and the perceived degree of importance of the items. All statistical analyses were performed using SPSS Version 24 (IBM Corporation, Armonk, NY, USA).

## Results

### Participants’ characteristics

Table 1 presents participants’ sociodemographic and clinical characteristics. Most (67.9%) were aged ≥50 years; had been diagnosed with BC between 41 and 50 years of age (60.7%); and had undergone BCS (65.1%), radiation therapy (62.7%) and/or hormone therapy (71.5%). About half (50.3%) preferred to make treatment decisions collaboratively with their doctors, while roughly one-third (31.3%) perceived that they collaborated with their doctors in choosing their treatment. Moreover, about half (51.4%) had some regret regarding the decision-making process.

**Table 1:** Participants’ sociodemographic and clinical characteristics (*N* = 467)

Characteristics	*n*	(%)
Age (mean (standard deviation))	53.4	(7.8)
Age group		
< 50 years	150	(32.1)
≥ 50 years	317	(67.9)
Age group at diagnosis		
≤ 40 years	87	(18.6)
41–50 years	253	(54.2)
> 50	127	(27.2)
Marital status		
Married	339	(72.6)
Other	128	(27.4)
Education		
≤ High school	122	(26.1)
> High school	345	(73.9)
Household income		
< ¥5,000,000	197	(42.2)
≥ ¥5,000,000	194	(41.5)
I do not know/ I do not want to share	76	(16.3)
Employment status		
Full time	99	(21.2)
Contract/ part–time	154	(33.0)
Others	214	(45.8)
Time since diagnosis		
≤ 5 years	256	(54.8)
> 5 years	211	(45.2)
Cancer stage		
0	72	(15.4)
I	220	(47.1)
II	175	(37.5)
Surgery type		
Breast conserving surgery	304	(65.1)
Total mastectomy	163	(34.9)
Radiation therapy		
No	174	(37.3)
Yes	293	(62.7)
Chemotherapy		
No	288	(61.7)
Yes	179	(38.3)
Hormone therapy		
No	133	(28.5)
Yes	334	(71.5)
Preferred decision-making role		
Active	178	(38.1)
Collaborative	235	(50.3)
Passive	54	(11.6)
Perceived decision-making role		
Active	229	(49.0)
Collaborative	146	(31.3)
Passive	92	(19.7)
Decisional congruence		
Incongruence	202	(43.3)
Congruence	265	(56.8)
Regret in treatment decision-making process		
Have none	227	(48.6)
Have some	240	(51.4)

### Correlates of regret in the decision-making process for cancer treatment

Table 2 presents the impact of sociodemographic and clinical variables, as well as decision-making roles, on the likelihood that patients would have some regret regarding the decision-making process, as revealed by multivariate logistic regression analyses. Sociodemographic correlates of regret in treatment decision-making were age upon being diagnosed with BC and employment status. Specifically, women diagnosed with BC when aged ≥50 years and who worked on a contract or part-time basis were significantly less likely to experience regret in the decision-making process than were their counterparts. In addition, only surgical type showed an association with regret in treatment decision-making: participants who received a TM were significantly more likely to feel regret regarding treatment decision-making compared with those who had BCS. Further, women who had TM and who made their decision for cancer treatment collaboratively with their doctors were significantly less likely to experience regret about the decision-making process compared with those who decided by themselves; women who had BCS and who participated in the process in their preferred role were significantly less likely to experience regret compared with those who did not participate in their preferred role.

**Table 2:** Multivariate logistic regression analysis concerning having regret in the treatment decision-making process

	All participants		BCS		TM	
Characteristic	AOR	95% CI	AOR	95% CI	AOR	95% CI
Age						
< 50 years	1	(ref)	1	(ref)	1	(ref)
≥ 50 years	0.95	(0.57–1.70)	0.65	(0.32–1.31)	1.60	(0.59–4.37)
Age at diagnosis						
≤ 40 years	1	(ref)	1	(ref)	1	(ref)
41–50 years	0.61	(0.33–1.10)	0.91	(0.42–1.98)	0.19	(0.06–0.61)**
> 50	0.44	(0.21–0.94)*	0.54	(0.20–1.45)	0.19	(0.04–0.87)*
Marital status						
Married	1	(ref)	1	(ref)	1	(ref)
Other	1.53	(0.96–2.44)	1.54	(0.86–2.74)	1.81	(0.77–4.23)
Education						
≤ High school	1	(ref)	1	(ref)	1	(ref)
> High school	1.13	(0.73–1.77)	1.03	(0.59–1.82)	1.46	(0.64–3.30)
Household income						
< ¥5,000,000	1	(ref)	1	(ref)	1	(ref)
≥ ¥5,000,000	1.11	(0.71–1.73)	1.00	(0.57–1.73)	1.40	(0.61–3.24)
I do not know/ I do not want to share	1.01	(0.57–1.77)	1.03	(0.51–2.09)	1.09	(0.38–3.13)
Employment status						
Full time	1	(ref)	1	(ref)	1	(ref)
Contract/ part–time	0.51	(0.29–0.89)*	0.41	(0.20–0.82)*	0.82	(0.29–2.28)
Others	0.64	(0.37–1.11)	0.64	(0.33–1.26)	0.74	(0.28–1.97)
Time since diagnosis						
≤ 5 years	1	(ref)	1	(ref)	1	(ref)
> 5 years	1.07	(0.69–1.67)	1.09	(0.63–1.90)	1.16	(0.49–2.74)
Cancer stage						
0	1	(ref)	1	(ref)	1	(ref)
I	0.94	(0.52–1.70)	0.95	(0.45–2.01)	0.79	(0.26–2.42)
II	0.80	(0.41–1.56)	0.75	(0.32–1.78)	0.77	(0.23–2.57)
Surgery type						
Breast conserving surgery	1	(ref)				
Total mastectomy	2.10	(1.11–3.98)*				
Radiation therapy						
No	1	(ref)	1	(ref)	1	(ref)
Yes	1.69	(0.90–3.17)	1.61	(0.70–3.69)	1.99	(0.68–5.87)
Chemotherapy						
No	1	(ref)	1	(ref)	1	(ref)
Yes	1.26	(0.79–2.01)	1.25	(0.70–2.24)	1.48	(0.63–3.45)
Hormone						
No	1	(ref)	1	(ref)	1	(ref)
Yes	1.15	(0.73–1.80)	1.30	(0.71–2.38)	0.88	(0.41–1.91)
Preferred decision-making role						
Active	1	(ref)	1	(ref)	1	(ref)
Collaborative	1.04	(0.65–1.68)	0.77	(0.42–1.40)	1.65	(0.67–4.06)
Passive	0.90	(0.42–1.93)	0.95	(0.37–2.45)	0.63	(0.15–2.67)
Perceived decision-making role						
Active	1	(ref)	1	(ref)	1	(ref)
Collaborative	0.54	(0.32–0.90)*	0.76	(0.40–1.43)	0.24	(0.09–0.68)**
Passive	0.99	0.53–1.84	0.99	(0.46–2.15)	1.12	(0.34–3.67)
Decisional congruence						
Incongruence	1	(ref)	1	(ref)	1	(ref)
Congruence	0.70	(0.45–1.10)	0.56	(0.32–0.98)*	1.13	(0.49–2.61)

*AOR* adjusted odds ratio, *CI* confidence interval, *ref*. reference group

*p < .05; **p < .001

Table 3 shows the most regrettable experiences regarding the decision-making process for women who reported some regret. The most frequent regrets were issues related to gathering information (23.8%) and consulting with others (21.3%).

**Table 3:** The most regrettable experiences among women who had some regret (*N* = 240)

Experiences	*n*	(%)
Matters that concern gathering information	57	(23.8)
Not consulting with others	51	(21.3)
Not considering which hospital to go to	37	(15.4)
Not taking the time to consider options	33	(13.8)
Not building trusting relationships with doctors	27	(11.3)
Other	35	(14.6)

### Importance of other factors on treatment decision

Table 4 presents the degree of importance of various factors when considering treatment options by participants’ age group. Cancer characteristics and treatment benefits and risks were very or fairly important for most participants, regardless of age. For women who were diagnosed with BC at a younger age, the potential impact on their sex life and pregnancy and childbirth was more important when making their medical decisions than for older women.

**Table 4:** Degree of importance of various factors related to decision-making by age at diagnosis

	Age at diagnosis (*n*, %)			
	≤ 40	41–50	> 50	*p*-value
Cancer characteristics				
Very or fairly important	80 (92.0)	238 (94.1)	118 (92.9)	.139
Important	7 (8.0)	10 (4.0)	9 (7.1)	
Slightly or not at all important	0 (0.0)	5 (2.0)	0 (0.0)	
Treatment benefits and risks				
Very or fairly important	81 (93.1)	237 (93.7)	121 (95.3)	.764
Important	6 (6.9)	16 (6.3)	6 (4.7)	
Slightly or not at all important	0 (0.0)	0 (0.0)	0 (0.0)	
Obtaining a second opinion				
Very or fairly important	33 (37.9)	80 (31.6)	45 (35.4)	.604
Important	33 (37.9)	99 (39.1)	42 (33.1)	
Slightly or not at all important	21 (24.1)	74 (29.2)	40 (31.5)	
Medical expenses				
Very or fairly important	55 (63.2)	152 (60.1)	70 (55.1)	.439
Important	16 (18.4)	65 (25.7)	37 (29.1)	
Slightly or not at all important	16 (18.4)	36 (14.2)	20 (15.7)	
Influence on pregnancy and childbirth				
Very or fairly important	28 (32.2)	16 (6.3)	7 (5.5)	<.001
Important	20 (23.0)	42 (16.6)	9 (7.1)	
Slightly or not at all important	39 (44.8)	195 (77.1)	111 (87.4)	
Influence on sex life				
Very or fairly important	24 (27.6)	18 (7.1)	8 (6.3)	<.001
Important	22 (25.3)	56 (22.1)	21 (16.5)	
Slightly or not at all important	41 (47.1)	179 (70.8)	98 (77.2)	
Influence on work and/or housework				
Very or fairly important	57 (65.5)	158 (62.5)	67 (52.8)	.337
Important	19 (21.8)	60 (23.7)	37 (29.1)	
Slightly or not at all important	11 (12.6)	35 (13.8)	23 (18.1)	

## Discussion

In our Internet survey of Japanese women with BC, approximately half of the women surveyed reported some regret in the treatment decision-making process. For one quarter of them, the most regrettable experience concerned the process of gathering information for their decision-making. Multiple logistic regression analyses revealed that the pattern of association between the decision-making and regret with the decision-making process differed by surgical type. For women who were diagnosed with BC at a relatively young age, the influence of treatment on their sex life and fertility was more important than it was for women who were older.

Our results suggested effective participation in the decision-making process differed by surgical type. For patients who had TM, making treatment decisions collaboratively with their doctors was associated with lower levels of regret; for patients who had BCS, congruence between preferred and perceived actual role was a predictor for not having regret. This result regarding TM contrasts with our previous finding that Japanese women with BC are more satisfied with treatment decision-making when their participation matches their preferred role, regardless of how they participate [11]. Differences in the cancer stage of the participants in the two studies may explain this inconsistency. Women who reported that they had stage 0–II BC were included in the current study, while women who reported that they had any stage of BC, including unknown stages, participated in the previous study. In the previous study, women who were diagnosed with stage III or IV BC were more likely to play a passive role in decision-making, and satisfaction with the decision-making process for women in this group was highest when their decisional role was congruent [11]. This is consistent with a study of Taiwanese women with early stage BC: regret regarding surgical decisions increased with decisional role incongruency, but only among women with stage II BC [7]. As the practice of shared decision-making has been recommended in oncological settings, questions have been raised as to whether this approach is beneficial to all patients. Our results provide insight into this question— collaborative decision-making may play a stronger role with regard to regret for those who had a higher stage of BC and who have undergone TM (data shown in Additional file 1: Table S1). Conversely, for patients who preferred to have BCS, who had a lower stage of cancer and who participated in decision-making in the manner in which they preferred may lead to less regrettable decision-making. Different approaches to decision-making based on their cancer stage or available surgical options may best serve the needs of BC patients.

Furthermore, type of surgery was the only clinical variable associated with decision-making regret. The AOR of having regret was significantly lower among women who had BCS than those who had TM. Similarly, the aforementioned study in Taiwan [7] and a population-based study in the U.S. [3] showed that regrets with decisions and the decision-making processes were lower among patients who had BCS compared with those who had TM. The most frequent factors associated with preferring TM among women with early stage BC in systematic review were survival and recurrence (46%) while it was 17% for those women who preferred BCS [13]. This suggests that patients in current study, who have higher stage of cancer may experience more difficulty in their decision-making process compared with patients who have lower stage of cancer due to their misconception regarding BC stage and the treatment options. Unnecessary concerns of death or cancer recurrence may take away their opportunities to participate in decision-making in the way they would prefer, resulting in them feeling not fully involved in the decision-making process. Additionally, the proportion of decisional incongruence in patients who played a passive role was higher for women with TM compared with those who underwent BCS (76% vs. 64%, respectively; data shown in Additional file 1: Table S2) suggests that some patients with TM may have unwillingly had this surgery as a result of their insufficient knowledge about their treatment options. These findings indicate that shared decision-making would be an appropriate approach to assist patients whose knowledge of BC is determined to be insufficient. On the other hand, this may not always be appreciated by patients who understand their eligibility and the equal efficacy of the two surgical options: TM and BCS with radiotherapy. For these women, healthcare professionals should verify how patients would like to be involved in their medical decision-making and provide guidance in this area.

Compared with women who were working full-time at the time of the survey, women who worked on a contract or part-time basis showed significantly lower odds of having decision-making regret. This was found only for women who had BCS. Treatment costs of BC increase as the stage of cancer increases [14] and cost is a major concern for patients with cancer everywhere, including Japan [15–17], often forcing patients to stay in their full- time job no matter what they truly desire. However, we do not know whether they continued their work after their diagnosis with BC. Further studies are needed to clarify the influence of the financial burden of cancer treatment on employment status, decision-making regret, and their interaction.

Given the extensive availability of BC treatments, a recommendation of active participation in medical decision-making may make some women feel overwhelmed. Among the women who reported regret in this study, nearly one-quarter indicated that their most regrettable experience concerned gathering information or not consulting with others. These results are consistent with other studies conducted in Japan [9, 18]. Nakashima and colleagues showed that more than half of their study participants with BC reported negative experiences with information-seeking, which includes a lack of available information, the time and effort required for finding information, and the lack of time to search [9]. Relatively recent data regarding the information that Japanese patients and survivors with BC sought on the largest cancer information website in Japan from April 2012 to December 2017 suggests that women were frustrated because they could not find the information they needed [18]. Next to information on metastasis and recurrence, treatment-related information such as possible treatments, hormone therapy, and the side effects of various treatments were the most frequently sought but most difficult to find information on this site [18]. Even when women could find the information they sought, they had concerns regarding its quality and had difficulties understanding the information they found [9]. This may be the reason why some women in the present study reported that their greatest regret regarding decision-making was that they did not consult with others.

According to the results of the multiple logistic regression analyses, women who perceived that they collaboratively made the treatment decision with their doctors were less likely to have regret about the process, suggesting that doctors are among those with whom they wish to consult. A German study of patients with breast or colon cancer showed that patients who perceived that their physicians showed them more sympathy were more likely to play a collaborative role in decision-making and have less decisional regret [8]. In a study of Chinese women with BC, a higher degree of shared decision-making at the diagnostic treatment consultation, which includes the provision of clear and unbiased information by the physician, was associated with greater satisfaction with the decision-making and less regret with the decision [5]. Sufficient time to discuss the type of cancer and its treatment and expressing to the doctor one’s preferences and feeling about the treatment choices, would produce less stressful decision-making, and may lead to greater satisfaction with the decision.

The present study revealed that age at diagnosis was associated with decision-making regret, especially for women who had TM. Women who had TM and were diagnosed with BC when they were aged older than 41 years were significantly less likely to experience regret in the decision-making process than were women who diagnosed with when they were younger than 40. In women aged younger than 40 years, the influence of BC treatment on pregnancy/childbirth and their sex life was considered more important when they chose their cancer treatment than it was for older women. This did not differ by surgery type (data shown in Additional file 1: Table S3). According to 2013 Japanese cancer statistics, among women under 30, BC accounted for less than 5% of cancer incidence; however, that percentage rises to over 20% for women in their thirties and is about 40% for women in their early forties [1]. Considering this statistic, our findings indicate that information regarding fertility and sexuality, is a critical factor in treatment decision-making for young patients.

Regarding fertility, as described earlier, a gap between the information that younger patients need and the information that health-care providers provide may exist. Two surveys targeting doctors who routinely examined patients with cancer including breast oncologists illustrate this gap. The proportion of doctors who reported that they referred patients who expressed fertility concerns to reproductive specialists was 42% in a national survey [19], and 30% in an Internet survey [20], and the proportion of doctors who routinely discussed fertility issues with their patients was 21 and 42.7%, respectively [19, 20]. The greatest barrier for physicians to discuss fertility issues with young patients was the priority of cancer treatment, especially for patients with aggressive disease [19, 20]. Time constraints in the clinic was a second greatest barriers for breast oncologist [19]. Therefore, bridging this gap by using non-physicians such as nurses, social workers and psychologists is anticipated [21].

In contrast to fertility, only a few small-sample studies have investigated the impact of BC on Japanese women’s sexuality [22, 23]. One such study, which involved 102 post-surgery patients, showed that the average age of patients who chose a skin-sparing mastectomy and immediate breast reconstruction was younger than were those who chose BCS or TM. Further, the value that these younger patients attached to their physical appearance, self-evaluation of femininity, and sexuality was significantly higher than for other surgery types [22]. Another study of 85 BC survivors, who were aged mainly in their forties or fifties and who had an active sexual relationship before surgery, showed that 73 of the women had resumed sex after surgery and that, while all except one reported sex-related symptoms, only nine of the women had consulted someone about their sexual concerns [23]. In addition, about half of the women in their twenties, thirties, or forties expressed a desire to include sexuality-related topics in a booklet for patients with BC [23]; for example, “influence of various treatment modalities on sexual response,” “relationship between sexual activities and recurrence of the disease,” and “informational resources regarding partnership or sexual issues.” Thus, it is possible that some discussion of fertility and sexuality with health care providers can reduce feelings of regret in decision-making, especially for younger patients. Further study to determine the association between the quality and quantity of time taken to discuss fertility and sexuality concerns in a clinical setting and regret or satisfaction with decision-making is needed.

There are several limitations to this study. First, given the nature of Internet-based, self-administered surveys, we were unable to evaluate the accuracy of the medical information provided, such as cancer stage and surgery type. However, previous study found that the proportion of correctly reported surgery type is 97.6% among patients with lumpectomy and 99.3% for mastectomy [24], suggesting that self-reported medical information is reliable. Second, participants who were registered with an online marketing research company and were willing to participate in surveys were recruited for this study. Selection bias thus limits the generalizability of our results. However, the Internet-based survey enabled us to recruit participants from a variety of regions and age groups; this is a study strength, as previous studies of decision-making among Japanese patients with BC were conducted in a single city with a small number of participants [9, 10]. Third, we did not investigate how regret regarding the decision-making process is associated with decision regret or satisfaction. Patients with BC have two contrary feelings which are greater satisfaction or less regret with their decisions, as well as some regret related to their decision-making process [3, 12]. To provide information leading to better interventions that foster patients’ satisfaction with their treatment, further study investigating the details of this association is needed.

## Conclusion

Approximately half of the Japanese women with BC in this study reported some regret in the treatment decision-making process. Effective participation in decision-making differed by surgical types. Additionally, women who are diagnosed with BC at a relatively young age may require additional information and support regarding their sex life and fertility after cancer treatment.

## Additional file

Additional file 1: **Table S1.** Surgery type by cancer stage (*N* = 467). **Table S2.** Decisional congruence by perceived decision-making role and surgery (*N* = 467). **Table S3.** Degree of importance of various factors related to decision-making at the time stratified by surgery type. (DOCX 40 kb)

## Data Availability

Datasets used and analyzed during the currents study are available from the corresponding author on reasonable request.

## Abbreviations

AOR: adjusted odds ratio
BC: breast cancer
BCS: breast-conserving surgery
TM: total mastectomy
