# Supplementary material for: Correlates of regret with treatment decision-making among Japanese women with breast cancer: results of an internet-based cross-sectional survey
Source: BMC Womens Health. 2019 Jul 2;19:86. doi: 10.1186/s12905-019-0783-5 (PMC6607591; doi:10.1186/s12905-019-0783-5)
Supplement: Supplementary file 1 — Table S1. Surgery type by cancer stage (N = 467). Table S2. Decisional congruence by perceived decision-making role and surgery (N = 467). Table S3. Degree of importance of various factors related to decision-making at the time stratified by surgery type. (DOCX 40 kb) [file 12905_2019_783_MOESM1_ESM.docx]

Additional file 1: Table S1: Surgery type by cancer stage (N = 467)

|  |  | Types of surgery (n, %) | |  |
| --- | --- | --- | --- | --- |
|  |  | BCS | TM | *p*-value |
| Total |  | 304 (100) | 163 (100) |  |
| Cancer stage | 0 | 48 (15.8) | 24 (14.7) | .009 |
|  | I | 157 (51.6) | 63 (38.7) |  |
|  | II | 99 (32.6) | 76 (46.6) |  |

Additional file 1 Table S2: Decisional congruence by perceived decision-making role and surgery (N = 467)

|  | All participants | | | BCS | | | TM | | |
| --- | --- | --- | --- | --- | --- | --- | --- | --- | --- |
|  | Perceived decision-making role (n, %) | | | | | | | | |
| Decisional congruence | Active | Collaborative | Passive | Active | Collaborative | Passive | Active | Collaborative | Passive |
| Incongruence | 111 (48.5) | 29 (19.9) | 62 (67.4) | 67 (47.9) | 19 (18.8) | 40 (63.5) | 44 (49.4) | 10 (22.2) | 22 (75.9) |
| Congruence | 118 (51.5) | 117 (80.1) | 30 (32.6) | 73 (52.1) | 82 (81.2) | 23 (36.5) | 45 (50.6) | 35 (77.8) | 7 (24.1) |
| Total | 33 (100) | 196 (100) | 146 (100) | 140 (100) | 101 (100) | 63 (100) | 89 (100) | 45 (100) | 29 (100) |

Additional file 3 Table S3: Degree of importance of various factors related to decision-making at the time stratified by surgery type

|  | Age at diagnosis (n, %) | | | | | |  |
| --- | --- | --- | --- | --- | --- | --- | --- |
|  | ≤ 40 | | 41–50 | | > 50 | | *p*-value |
|  | BCS | TM | BCS | TM | BCS | TM |  |
| Cancer characteristics |  |  |  |  |  |  |  |
| Very or fairly important | 53 (90.0) | 27 (96.0) | 152 (95.0) | 86 (92.5) | 80 (94.0) | 38 (90.0) | BCM=.137  TM=.602 |
| Important | 6 (10.0) | 1 (3.6) | 5 (3.1) | 5 (5.4) | 5 (6.0) | 4 (10.0) |  |
| Slightly or not at all important | 0 (0.0) | 0 (0.0) | 3 (1.9) | 2 (2.2) | 0 (0.0) | 0 (0.0) |  |
| Treatment benefits and risks |  |  |  |  |  |  |  |
| Very or fairly important | 54 (82.0) | 27 (96.1) | 162 (95.0) | 85 (91.4) | 82 (96.0) | 39 (93.0) | BCM=.416  TM=.669 |
| Important | 5 (8.0) | 1 (4.0) | 8 (5.0) | 8 (8.6) | 3 (4.0) | 3 (7.0) |  |
| Slightly or not at all important | 0 (0.0) | 0 (0.0) | 0 (0.0) | 0 (0.0) | 0 (0.0) | 0 (0.0) |  |
| Obtaining a second opinion |  |  |  |  |  |  |  |
| Very or fairly important | 24 (41.0) | 9 (32.0) | 49 (30.6) | 31 (33.3) | 32 (38.0) | 13 (31.0) | BCM=.539  TM=.934 |
| Important | 21 (36.0) | 12 (43.0) | 62 (38.8) | 37 (39.8) | 27 (32.0) | 15 (36.0) |  |
| Slightly or not at all important | 14 (24.0) | 7 (25.0) | 49 (30.6) | 25 (26.9) | 26 (31.0) | 14 (33.3) |  |
| Medical expenses |  |  |  |  |  |  |  |
| Very or fairly important | 39 (66.0) | 16 (57.0) | 98 (61.3) | 54 (58.1) | 50 (59.0) | 20 (48.0) | BCM=.526  TM=.934 |
| Important | 11 (19.0) | 5 (18.0) | 46 (28.8) | 19 (20.4) | 24 (28.0) | 13 (31.0) |  |
| Slightly or not at all important | 9 (15.0) | 7 (25.0) | 16 (10.0) | 20 (21.5) | 11 (13) | 9 (21.0) |  |
| Influence on pregnancy and childbirth |  |  |  |  |  |  |  |
| Very or fairly important | 21 (36.0) | 7 (25.0) | 9 (5.6) | 7 (7.5) | 4 (5.0) | 3 (7.0) | BCM= <.001  TM=.004 |
| Important | 13 (22.0) | 7 (25.0) | 24 (15.0) | 18 (19.4) | 7 (8.0) | 2 (5.0) |  |
| Slightly or not at all important | 25 (42.0) | 14 (50.0) | 127 (79.4) | 68 (73.1) | 74 (87.0) | 37 (88.0) |  |
| Influence on sex life |  |  |  |  |  |  |  |
| Very or fairly important | 15 (25.0) | 9 (32.0) | 10 (6.3) | 8 (6.6) | 5 (5.9) | 3 (7.1) | BCM= <.001  TM=.005 |
| Important | 17 (29.0) | 5 (18.0) | 36 (22.5) | 20 (21.5) | 16 (19.0) | 5 (12.0) |  |
| Slightly or not at all important | 27 (46.0) | 14 (50.0) | 114 (71.3) | 65 (69.9) | 64 (75.0) | 34 (81.0) |  |
| Influence on work and/or housework |  |  |  |  |  |  |  |
| Very or fairly important | 37 (63.0) | 20 (71.0) | 102 (63.8) | 56 (60.2) | 43 (51.0) | 24 (57.0) | BCM=.332  TM=.790 |
| Important | 14 (24.0) | 5 (18.0) | 39 (24.4) | 21 (22.6) | 26 (31.0) | 11 (26.0) |  |
| Slightly or not at all important | 8 (14.0) | 3 (11.0) | 19 (11.9) | 16 (17.2) | 16 (19.0) | 7 (17.0) |  |
